# Supplementary material for: MetaRibo-Seq measures translation in microbiomes
Source: Nat Commun. 2020 Jun 29;11:3268. doi: 10.1038/s41467-020-17081-z (PMC7324362; doi:10.1038/s41467-020-17081-z)
Supplement: Supplementary file 10 — Supplementary Data 7 [file 41467_2020_17081_MOESM10_ESM.zip › File2/Confidence_High_Taxonomy/396518_out.krona.html]

Javascript must be enabled to view this page.

members
magnitude
magnitudeUnassigned
count
unassigned
taxon
rank

396518\_out

6

6
superkingdom
2

6
phylum
1239

6
class
186801

order
186802
6

3
family
186803

3
1
genus
572511

SRS149181\_contig\_number\_contig-100\_11084.131190


SRS015217\_contig\_number\_contig-100\_15127.111798
species
765821
1

1226324

SRS143991\_contig\_number\_16322
species
1

family
541000
3

1263
genus
3

1

SRS077502\_contig\_number\_7726
species
657323

1
species

SRS148511\_contig\_number\_7595
1262968


SRS050422\_contig\_number\_14337
species
2293208
1
